# Supplementary material for: Primed Track, high-fidelity lineage tracing in mouse pre-implantation embryos using primed conversion of photoconvertible proteins
Source: eLife. 2019 Jan 21;8:e44491. doi: 10.7554/eLife.44491 (PMC6340703; doi:10.7554/eLife.44491)
Supplement: Figure 2—source data 1. [file elife-44491-fig2-data1.docx]

| Analysis challenges in drifting pre-implantation embryos | TGMM | Ilastik | TrackMate |
| --- | --- | --- | --- |
| Segmentation | - Cells do not get segmented once cells density increases | - Segmentation breaks cells into multiple segments - Background signal is also segmented | - Cells do not get properly segmented in each time frame |
| Tracking | - Cells are connected to wrong tracks due to spatial / rotational drift as well as the large cell division place in early embryos (i.e. daughter cells are positioned far from their mother after division) | - Cells lose their original identify and are connected to wrong tracks in consecutive time points - Background signal far from the embryo can be given the same ID as cells within the embryo | - Cells lose their original identify and are connected to wrong tracks in consecutive time points |
| Lineage tracing | - Highly disorganized lineage trees for drifting embryos | - No automatic reconstruction of a lineage tree from tracked cells - Highly disorganized lineage trees for drifting embryos | - Difficult to infer which cells in the imaging data correspond to individual tracks in the lineage trees - Highly disorganized lineage trees for drifting embryos |
